# The Common Task

**Published:** 1907-05-04

**Authors:** 


### THE COMMON TASK.

**Correspondence and Queries** for this section should be sent to the Editor of The Hospital, 28 Southampton Street, Strand, London, and marked "Nursing Administration."

### Dangers of Over-training.

While certain New York hospitals appear to be taking the retrograde step of reducing the course of training in their wards for pupil nurses to two years, a feeling is growing in the minds of many medical men that the curriculum prescribed by some enthusiastic American superintendents is far too comprehensive, even for the more extended course of three years. Dr. Bristow gives expression to this feeling in a paper read before the New York State Nurses' Association, in which he humourously declares that "the nurse who survives this course will be a chemist, a physiologist, a cook, and a confectioner." The fact is that long experience in the technical business of examining is necessary to fit people, however skilled in their own department, to set a curriculum. Every branch of knowledge is practically limitless, and the crux of the matter in all education is what to omit. What to include is a comparatively easy matter to decide. In a curriculum built up out of the practical experience of a committee of nurses it would be found to include every subject which any individual member had found of use to herself. The training of nurses demands before everything else sincerity and thoroughness, and the subjects taught must be few enough to ensure their complete mastery by women of average ability.

### Discipline Among the Babies.

Dr. George Armstrong, who instituted a dispensary for the relief of the infant poor in 1769, and was thus pioneer of the modern children's hospital, had small expectation that in-patients could ever be received in such establishments. "If you take away a sick child from its parent or nurse, you break its heart immediately," he pleads, "and if there must be a nurse to each child, what kind of hospital must there be to contain any number of them? Would not mothers and nurses be perpetually at variance?" His foreboding words were in our mind when visiting lately the babies' ward of the Royal Berkshire Hospital, in which twelve people, between the ages of one and five, were behaving like sages under the eye of two nurses. Driven in by boisterous breezes from their balcony, they were all in a lively mood, but there was not one of them who had not learned the wonderful lesson of discipline which flourishes imperceptibly in a well-managed ward, and develops in a child, even before he can speak, the social instinct of trying to please instead of trying to annoy. If only district nurses could impart this subtle art of discipline to the mothers of these babes, we should no longer hear, as we did the other day, of the mother who, when told that she ought to wean her fifteen months old child, complained that "the baby won't be weaned." It would astonish most people to learn how many babies die every year solely from getting their own way.

### Beef-tea Extracts.

Mr. A. L. Wauchope Watson, author of "Physiological Aspects of Dietary," writes:—To accept as a postulate the statement that "the price of freshly-made beef-tea should not be more than 2d. a pint at the outside" would, if correct, effectively strangle any argument as to the economy of meat extracts over those preparations freshly made from beef. In no single hospital, in London, however, do I know of a preparation obtained at such a figure, by reason of the fact that the average contract price of beef used for this purpose is between 4d. and 5d. per lb. In the making of beef-tea, then, it is assumed that 1 lb. of beef is used to a pint of water, the custom being one adhered to by the vast majority of nursing institutions where a preference is given to the old-fashioned mode of manufacture. In a few isolated cases, however, I have known half a pound of beef to be used to a pint of water, but analytical determinations have shown these preparations to be practically worthless. Thus to arrive at a satisfactory conclusion as to the true economy of meat extracts over freshly made beef-tea it is necessary to take a given quantity of extract of meat sufficient to make the same quantity of the former preparation and to carefully compare the analyses of both. For example, to 1 lb. of Bovril, costing 4s. 6d., add 21 pints of water as laid down by the manufacturers' directions and compare the same with an equal quantity of ordinary beef-tea. I submit then that a considerable saving will be shown, and when the cost of preparing ordinary beef-tea is taken into account, including fuel, labour, etc., the obvious economy of such substitutes for beef-tea is evident. In addition, the albumen and fibrine present in this preparation give it an advantage over and above the saving of labour and of expense.

. ˙. Excellent foreign shin of beef can be obtained in bulk at a price of from 2½d. to 3½d. per lb. If the recipe given in "Hospital Expenditure: The Commissariat," page 113, be followed, three-quarters of a pound of meat to each pint will be found to give good tea. "Mince the meat small, including all fat and gristle, pour on about three-quarters of the prescribed quantity of water cold, and soak for one hour; let it simmer gently for three or four hours in a covered jar or saucepan, never actually boiling, though near boiling-point, and then pour off without straining, since much of the food-value is contained in the floating brown particles. To the rags of meat at the bottom of the vessel add the remaining proportion of water hot, and let it simmer for an hour or two. Pour off and add to the tea already infused, and the brew will be superior in strength to that made in the proportion of one pound to a pint." We propose to deal with this matter in more detail shortly.—Ed., The H.

